# *Notes From the Field:* First Evidence of Locally Acquired Dengue Virus Infection — Maricopa County, Arizona, November 2022

**DOI:** 10.15585/mmwr.mm7211a5

**Published:** 2023-03-17

**Authors:** Melissa Kretschmer, Jennifer Collins, Ariella P. Dale, Brenna Garrett, Lia Koski, Karen Zabel, R. Nicholas Staab, Katie Turnbow, Judah Nativio, Kelsey Andrews, William E. Smith, John Townsend, Nicole Busser, James Will, Kathryn Burr, Forrest K. Jones, Gilberto A. Santiago, Kelly A. Fitzpatrick, Irene Ruberto, Kathryn Fitzpatrick, Jessica R. White, Laura Adams, Rebecca H. Sunenshine

**Affiliations:** ^1^Maricopa County Department of Public Health, Phoenix, Arizona; ^2^Maricopa County Environmental Services Vector Control, Phoenix, Arizona; ^3^Arizona Department of Health Services; ^4^Epidemic Intelligence Service, CDC; ^5^Division of Vector-Borne Diseases, National Center for Emerging and Zoonotic Infectious Diseases, CDC; ^6^Career Epidemiology Field Officer Program, CDC.

On November 7, 2022, dengue virus (DENV), which is not endemic in the continental United States (*1*), was identified in a Maricopa County, Arizona resident by reverse transcription–polymerase chain reaction (RT-PCR) testing at Arizona State Public Health Laboratory (ASPHL). The patient (patient A) was admitted to a hospital on October 19 for a dengue-like illness, 7 days after traveling to and remaining in Mexicali, Mexico for <4 hours. Patient A was hospitalized for 3 days and subsequently recovered. Maricopa County Environmental Services Department (MCESD) conducted retrospective testing for DENV in samples collected from 21 mosquito pools located within 5 miles (8 km) of patient A’s residence during October 1–November 3. A sample collected from one mosquito pool (pool A) on October 5 was positive for DENV. Whole genome sequencing by CDC’s Dengue Branch later revealed that closely related DENV-3 strains not known to be circulating in the patient’s travel region were identified in both patient A and pool A, suggesting local DENV transmission.

Based on a preexisting, joint Maricopa County Department of Public Health (MCDPH), MCESD, and Arizona Department of Health Services locally acquired mosquitoborne disease response plan, MCDPH and MCESD activated an incident command office on November 10. MCDPH took the following actions: 1) prioritized prospective investigations of health care provider and laboratory reports of DENV and suspected arboviral visits queried from the National Syndromic Surveillance Program's BioSense Platform (BioSense)*; 2) retrospectively reviewed confirmed, probable, and suspected dengue cases investigated during July 1–November 10 for evidence of local DENV transmission; 3) alerted health care providers of the possible local transmission; and 4) advised providers to test for and report suspected DENV to MCDPH. No evidence of local acquisition was identified in 13 suspected arboviral visits identified in the Biosense database, 10 reviews of closed cases, and 10 new case investigations. MCESD retrospectively tested samples collected during September 18–November 19 from an additional 4,299 mosquito pools located throughout the county, including the mosquito pools within 5 miles of patient A’s residence collected during the expanded testing time frame, for DENV by RT-PCR; all were negative. This activity was reviewed by CDC and was conducted consistent with applicable federal law and CDC policy.^†^

After discussions with CDC’s Dengue Branch and Florida Public Health (*2*,*3*) regarding current best practices for managing locally acquired DENV infections, during November 17–19, MCDPH and MCESD canvassed residences within a 0.09-mile (150-m) radius (*4*) of patient A’s residence and pool A to interview residents, collect human specimens for DENV testing, and assess properties for mosquito breeding. Teams approached 241 households; residents of 72 households (29.9%) consented to environmental assessments, and 73 persons in 59 (24.5%) households were interviewed. Among these 73 interviewees, 12 (16.4%) reported onset of dengue-like symptoms within 14 days of their interview and received testing; all results were negative for DENV by RT-PCR at ASPHL. A serum enzyme immunoassay for DENV immunoglobulin M testing was performed by ASPHL on blood specimens from 53 (72.6%) interviewees; among these, one (1.9%) result was positive. CDC Arboviral Diseases Branch confirmed DENV-3 by plaque reduction neutralization testing. The person with the positive test result reported no travel during the 2 weeks preceding symptom onset. One of this person’s household members reported dengue-like symptoms but declined testing; both have since recovered. Environmental assessment of this residence identified *Aedes aegypti* mosquitoes and breeding sites; mosquitoes collected in a professional BG-Sentinel mosquito trap^§^ tested negative for DENV by RT-PCR. The outbreak (consisting of two autochthonous DENV infections) ended January 4, 2023, after >45 days without additional locally acquired cases, as indicated by most recent guidance (*4*).

Coordinated surveillance and response activities identified the first locally acquired human DENV infections in Maricopa County, Arizona. Established partnerships and preexisting plans were essential to mounting a rapid, coordinated response to nonendemic arboviral transmission. MCDPH and MCESD will enhance future surveillance activities to identify and prevent autochthonous DENV transmission, including additional mosquito trap placement around patient residences and public mosquito exposure prevention education.^¶^ A countywide health care provider education campaign is being implemented to increase provider awareness of local DENV transmission and encourage testing for patients with compatible illness, irrespective of travel history.

## Footnotes

*
https://www.cdc.gov/nssp/overview.html

† 45 C.F.R. part 46, 21 C.F.R. part 56; 42 U.S.C. Sect. 241(d); 5 U.S.C. Sect. 552a; 44 U.S.C. Sect. 3501 et seq.

§
https://www.bg-sentinel.com/

¶
www.maricopa.gov/fightthebite
